# Regional differences in BMI, obesity, and exercise frequency in a large veteran service organization: A secondary analysis of new veteran member surveys from Team Red, White & Blue

**DOI:** 10.1016/j.pmedr.2018.09.001

**Published:** 2018-09-07

**Authors:** Justin T. McDaniel, Kate H. Thomas, Caroline M. Angel, Michael S. Erwin, Louis P. Nemec, Brandon B. Young, Nicholas J. Armstrong, Blayne P. Smith, John M. Pinter

**Affiliations:** a College of Education and Human Services, Department of Public Health and Recreation Professions, Southern Illinois University, 475 Clocktower Drive, Mailcode 4632, Carbondale, IL 62901, United States of America; b College of Health Sciences, 9200 University Blvd., Charleston, SC 29406, United States of America; c Team Red, White, and Blue, Institute for Veterans and Military Families, Syracuse University, 1110 W. Platt Street, Tampa, FL 33606, United States of America; d Team Red, White, and Blue, The Positivity Project, Pinehurst, NC 28374, United States of America; e Team Red, White & Blue, 1110 W. Platt Street, Tampa, FL 33606, United States of America; f Team Red, White & Blue, The Tennyson Center for Children, 2950 Tennyson Street, Denver, CO 80212, United States of America; g The Institute for Veterans and Military Families, Syracuse University, 150 Crouse Drive, Syracuse, NY 13244, United States of America; h Team Red, White, and Blue, 1110 W. Platt St., Tampa, FL 33606, United States of America; i Team Red, White, and Blue, 1110 W. Platt Street, Tampa, FL 33606, United States of America

**Keywords:** Obesity, Body mass index, Region, Veterans, Exercise

## Abstract

The purpose of the present study was to examine regional differences in average self-reported BMI, obesity prevalence, and frequent exercise (FE) among members of Team Red, White, and Blue (Team RWB) – a military veteran service organization founded to increase physical activity in veterans. A total of 10,015 military veterans participated in a needs assessment conducted by Team RWB between December 2014 and August 2016. Multivariate regression analysis with bootstrapped coefficients revealed that: BMI was highest in the Midwest region (*M* = 28.282) of the United States, *F*_(20, 9882)_ = 105.560, *p* < 0.001; obesity prevalence was highest in the Southcentral (32.300%) and Southeast (32.200%) regions, *x*^*2*^_(9731)_ = 10,850, *p* < 0.001; and FE was most prevalent in the Mid-Atlantic region (67.3%), *x*^*2*^_(9882)_ = 11,291, *p* < 0.001.The results of this study closely mirror results found in studies of the general population. A better understanding of the geographic distribution of these outcomes could guide the targeting of sub-populations for public health programs. In particular, Team Red, White & Blue community growth and other fitness based public health programs could be expanded to reach more veterans.

## 1 Introduction

Obesity is a public health problem in the U.S. (Hedley et al., 2004). Although obesity rates in the active-duty military are lower than in the general population, veterans are at similar risk to civilians (Almond et al., 2008). In veterans and non-veterans, physical inactivity and poor diet are associated with obesity (Nelson, 2006). Among the general population, obesity varies by region – with the South and Midwest exhibiting the highest prevalence (Myers, 2015). Little is known about regional differences in obesity among veterans. The purpose of this study was to examine body mass index (BMI), obesity, and exercise frequency by region among military veterans who self-selected participation in a veterans' service organization. A better understanding of the geographic distribution of these outcomes could guide the targeting of sub-populations for public health programs. In particular, Team Red, White & Blue community growth and other fitness based public health programs could be expanded to reach more veterans. We explored the following non-directional, alterative hypotheses:

1. Military veteran BMI differs by region of the United States.
2. Military veteran obesity prevalence rates differ by region of the United States.
3. Engagement in frequent exercise among military veterans differs by region of the United States.

## 2 Methods

### 2.1 Study design

Team RWB is a 501(c) organization focused on enriching veterans' lives through physical fitness and social activities to address reintegration challenges and offset physical health declines and weight gain following military to civilian transition (Angel et al., 2018). Since inception in 2010, Team RWB has grown to 209 chapters and 140,000 members, representing an intergenerational community of 70% veterans and 30% civilians (Team Red, White, and Blue, 2018a). The organization's reach is geographically-impactful and, in 2017, culminated in 24,939 exercise events, 6577 athletic events, 5633 social events, 2688 service events, and 7169 one-to-one member engagements (Team Red, White, and Blue, 2018b).

Team RWB staff collected baseline assessments of new members in 2014. Team RWB staff/researchers developed an instrument for the assessment. The instrument was reviewed by subject-matter experts and pilot-tested for completion time and psychometric properties. The final version of the online survey was offered to all new Team RWB members with a t-shirt incentive. Between December 2014–August 2016, 37,229 active-duty service-members, National Guard, reserve personnel, or veterans joined Team RWB. During this time-frame, 19,218 military-connected participants (52%) completed the survey. In this study, only veterans were analyzed (*n* = 10,015).

### 2.2 Dependent variables

Three dependent variables were examined: BMI, obesity (OB), and frequent exercise (FE). BMI was calculated with self-reported height and weight (BMI = [(weight(lbs) / height(in))^2^ ∗ 703]) (CDC, 2018). Three respondents exhibited BMIs <5 and were, thus, discarded. OB was based on the NHLBI's guideline for BMI categories: BMIs ≥30 were coded as obese (NHLBI, 2018). FE was based on one Likert-scale item: “I exercise frequently.” Response options included strongly disagree, disagree, neutral, agree, and strongly agree. “Strongly agree” and “agree” responses were coded as FE (Code = 1) while other responses were given a code of zero.

### 2.3 Independent variables

**Fig. 1:**
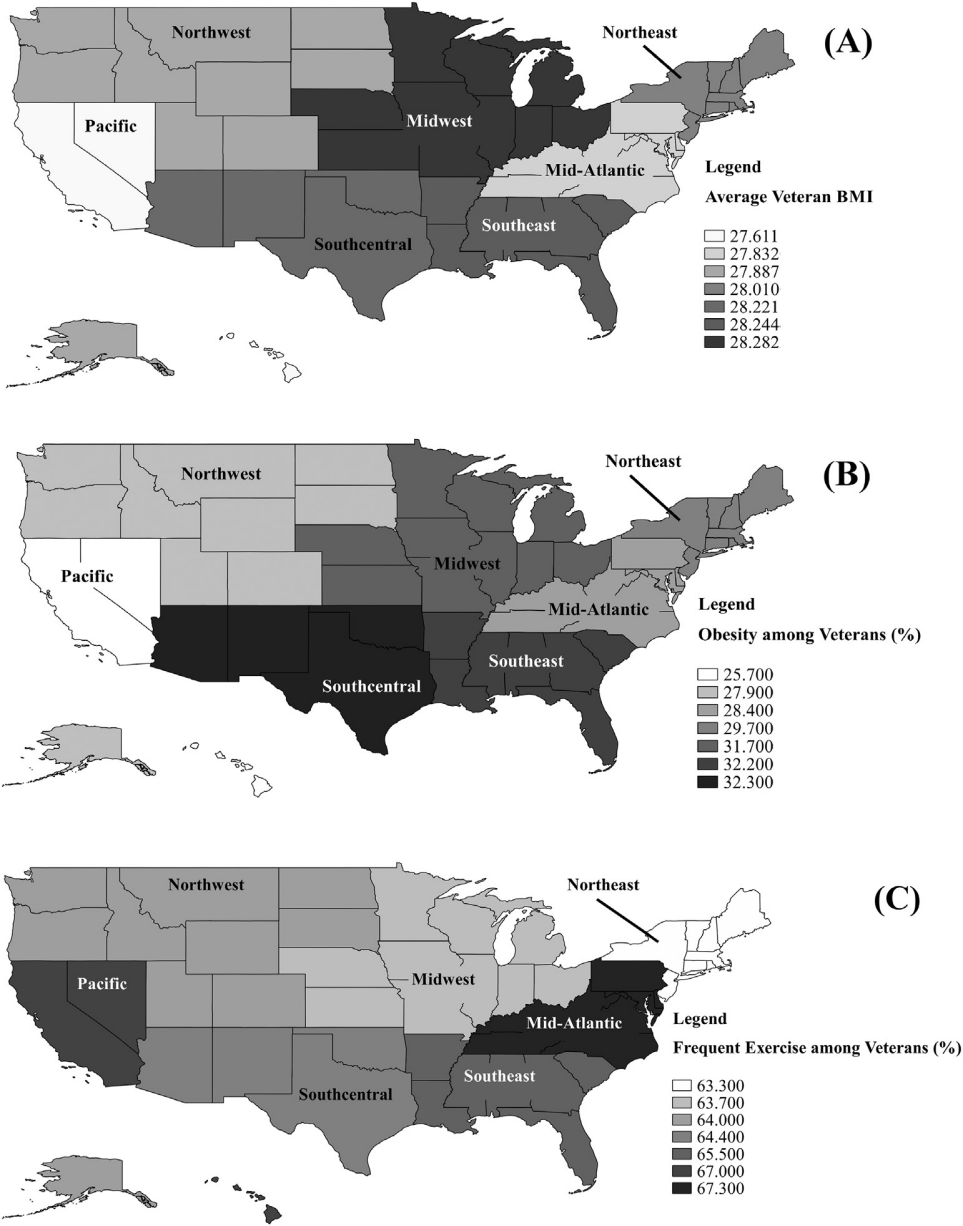
Maps of regional differences in (a) BMI, (b) obesity prevalence, and (c) frequent exercise prevalence among veterans between December 2014 and August 2016.

The primary independent variable was Team RWB region. Team RWB stratifies states into seven regions (Fig. 1): Northwest, Pacific, Southcentral, Midwest, Southeast, Mid-Atlantic, and Northeast. Respondents of the Team RWB survey were asked to provide their zip-code, which was used to code respondents into Team RWB regions (Team Red, White, and Blue, 2018a, Team Red, White, and Blue, 2018b).

### 2.4 Control variables

To isolate the effect of region on BMI, OB, and FE, and in keeping with other similar studies of veterans (Almond et al., 2008; Vieweg et al., 2006), several control variables were included. Items measuring race/ethnicity were omitted from the survey; therefore, we were unable to control for these factors. Gender was measured dichotomously (Female = 1, Male = 0). Employment status was measured with the following categories: employed, retired, disabled, and unemployed. Receipt of a “purple heart” – which was included as a proxy measure of previous traumatic experience – during military service was measured dichotomously (Yes = 1, No = 0). Use of Veterans Affairs (VA) healthcare benefits – which has been shown to be associated with higher BMI (Wang et al., 2012) – was measured dichotomously (Yes = 1, No = 0). Service era at the time of original entry – which served as a proxy for age – was measured with three categories: Operation Iraqi Freedom/Operation Enduring Freedom (OIF/OEF), Gulf war, and Vietnam/Korean war.

One item assessed anxious mood: “In the past month, I have felt nervous, anxious, or on edge.” One item assessed depressed mood: “In the past month, I have felt down, depressed, or hopeless.” For these constructs, a five-point response scale was offered: strongly disagree to strongly agree. “Strongly agree” and “agree” responses were grouped together (Code = 1) and all other responses were grouped together (Code = 0). Survey completion year was also controlled for in the present study to rule out historical effects.

Daily tobacco use was measured with the following question: “How would you characterize your use of tobacco products?” The following response options were available: never, once a month or less, once a week or less, weekly, daily, and frequently during the day. “Daily” or “frequently during the day” responses were grouped together (Code = 1) and all other responses were grouped together (Code = 0). Daily alcohol use was measured with the following question: “How would you rate your consumption of alcohol?” The following response options were available: never, once a month or less, once a week or less, occasional drinks during the week, every day, and more than a couple drinks every day. Responses of “every day” or “more than a couple drinks every day” were grouped together (Code = 1) and all other responses were grouped together (Code = 0).

### 2.5 Data analysis

Descriptive statistics for BMI, OB and FE were calculated according to the independent and control variables. Maps were created in QGIS (Quantum GIS Development Team, 2018) in order to visually represent regional differences in BMI, OB, and FE. Three regression models were estimated in this study. Robust linear regression with M-estimation was used to examine the effect of region on BMI (Huber, 1981). Maximum likelihood logistic regression (Hosmer and Lemeshow, 2005) was used to examine (a) the effect of region on OB, and (b) the effect of region on FE. The control variables listed earlier in this paper were included in all models. Beta coefficients in all models were bootstrapped with 1999 resamples (Shorack, 1982) and bias-corrected 95% confidence intervals were generated for each estimate (DiCiccio and Romano, 1988). Effect sizes (Cohen, 1988) were calculated for pairwise regional differences in BMI (Cohen's *d*), OB (Cohen's *h*), and FE (Cohen's *h*) based on significant results of the regression models.

## 3 Results

Average BMI was highest in the Midwestern region (Table 1, Fig. 1), while the lowest average BMI was evident in the Pacific region; however, results showed that all regions had an average BMI within the overweight BMI category, as established by the U.S. Department of Health and Human Services (NHLBI, 2018). Obesity prevalence was highest in the Southcentral and Southeast regions (Fig. 1). The Pacific region had the lowest proportion of obesity. FE was most common in the Mid-Atlantic region, while FE was least common in the Northeast (Fig. 1).

**Table 1:** Descriptive statistics for all study variables by BMI, obesity, and frequent exercise, December 2014 through August 2016.

	Sample Size *n* (%)	BMI^b^ *M* (*SD*)	Obese *n* (%)	Frequent exercise *n* (%)
Region				
Northwest	877 (8.800)	27.887 (4.926)	239 (27.900)	561 (64.000)
Pacific	766 (7.600)	27.611 (4.759)	192 (25.700)	513 (67.000)
Southcentral	1698 (17.000)	28.221 (4.867)	534 (32.300)	1094 (64.400)
Midwest	1856 (18.500)	28.282 (5.016)	575 (31.700)	1182 (63.700)
Southeast	1749 (17.500)	28.244 (5.154)	544 (32.200)	1146 (65.500)
Mid-Atlantic	1928 (19.300)	27.832 (4.858)	531 (28.400)	1298 (67.300)
Northeast	998 (10.000)	28.010 (4.775)	292 (29.700)	632 (63.300)

Gender				
Male	6863 (68.500)	28.764 (4.661)	2240 (33.400)	4463 (65.000)
Female	3152 (31.500)	26.486 (5.077)	696 (22.800)	2045 (64.900)

Employment status				
Employed	6943 (70.100)	27.944 (4.718)	1981(29.000)	4803 (68.500)
Retired	831 (8.400)	28.427 (5.105)	265 (32.100)	548 (64.900)
Disabled	577 (5.800)	29.968 (6.130)	258 (45.000)	240 (40.700)
Unemployed	1552 (15.700)	27.616 (4.966)	432 (28.300)	917 (58.600)

Received purple heart				
No	9731 (97.200)	28.025 (4.920)	2838 (30.000)	6347 (65.200)
Yes	284 (2.800)	28.923 (4.452)	98 (35.000)	161 (56.700)

Receive VA health benefits				
No	4131 (41.200)	27.393 (4.524)	999 (24.900)	3011 (72.900)
Yes	5884 (58.800)	28.513 (5.114)	1937 (33.800)	3497 (59.400)

Service era				
OIF/OEF^c^	2511 (25.600)	27.304 (4.732)	614 (24.800)	1611 (63.400)
Gulf War	6072 (61.900)	28.348 (4.920)	1928 (32.200)	4019 (65.500)
Vietnam/Korean War	1226 (12.500)	28.052 (5.067)	362 (30.100)	822 (66.200)

Anxious mood^a^				
No	5369 (53.600)	27.536 (4.539)	1345 (25.800)	3902 (72.700)
Yes	4646 (46.400)	28.644 (5.242)	1591 (35.000)	2606 (56.100)

Depressed mood^a^				
No	6840 (68.300)	27.578 (4.591)	1741 (26.200)	4890 (71.500)
Yes	3175 (31.700)	29.069 (5.395)	1195 (38.600)	1618 (51.000)

Daily tobacco use				
No	8992 (89.800)	27.985 (4.883)	2593 (29.600)	6026 (67.000)
Yes	1023 (10.200)	28.632 (5.104)	343 (34.500)	482 (47.100)

Daily alcohol use				
No	9626 (96.100)	28.064 (4.925)	2834 (30.200)	6278 (65.200)
Yes	389 (3.900)	27.736 (4.502)	102 (26.600)	230 (59.100)

Survey completion year				
2014	123 (1.200)	27.827 (4.806)	38 (30.900)	90 (73.200)
2015	5948 (60.100)	27.935 (4.842)	1706 (29.100)	3911 (65.000)
2016	3832 (38.700)	28.239 (5.012)	1192 (31.700)	2507 (64.700)

a Anxious mood and depressed mood measurements were taken between December 2014 – August 2016.

b BMI = body mass index.

c Operation Iraqi Freedom/Operation Enduring Freedom.

The overall BMI model (Table 2) was statistically significant, *F*_(20, 9882)_ = 105.560, *p* < 0.001. Veterans in the Northeastern, Southeastern, Midwestern, and Southcentral regions had significantly higher BMIs than veterans in the Pacific region. Cohen's *d* was calculated for the aforementioned statistically significant regional differences in order to determine the practical difference in means: Midwest vs. Pacific, *d* = 0.139; Southeast vs. Pacific, *d* = 0.128; Southcentral vs. Pacific, *d* = 0.127; and Northeast vs. Pacific, *d* = 0.084. Covariates associated with higher BMI included being male, having VA health care benefits, exercising infrequently, reporting symptoms consistent with depressed mood, not using alcohol/cigarettes daily, having served in the Gulf/Vietnam/Korean war eras, and being disabled.

**Table 2:** Regional differences in BMI, obesity prevalence, and frequent exercise among military veterans between December 2014 and August 2016.

	Model: BMI^c^, ^d^				Model: Obesity^e^				Model: Exercise^f^			
			95% BCa CI^b^				95% BCa CI^b^				95% BCa CI^b^	
Variable	b^b^	SE^b^	Lower	Upper	b^b^	SE^b^	Lower	Upper	b^b^	SE^b^	Lower	Upper
Intercept	−3.601	2.648	−9.008	1.487	−156.231	92.640	−312.012	62.845	68.366	92.537	−108.647	246.038
Region^a^												
Northwest	0.004	0.003	−0.002	0.010	0.145	0.115	−0.108	0.342	0.151	0.102	−0.049	0.354
Pacific	(Ref)				(Ref)				0.288⁎	0.110	0.074	0.510
Southcentral	0.010⁎	0.003	0.005	0.015	0.387⁎	0.099	0.187	0.584	0.157	0.088	−0.019	0.332
Midwest	0.008⁎	0.003	0.003	0.014	0.330⁎	0.098	0.133	0.523	0.159	0.085	−0.002	0.323
Southeast	0.010⁎	0.003	0.005	0.016	0.388⁎	0.099	0.205	0.595	0.283⁎	0.088	0.116	0.462
Northeast	0.007⁎	0.003	0.002	0.013	0.230⁎	0.111	0.004	0.441	(Ref)			
Mid-Atlantic	0.004	0.003	−0.001	0.011	0.242⁎	0.099	0.038	0.419	0.212⁎	0.086	0.043	0.379
Female	−0.039⁎	0.002	−0.042	−0.036	−0.589⁎	0.054	−0.695	−0.479	−0.391⁎	0.053	−0.496	−0.287
Purple heart	−0.001	0.004	−0.008	0.006	−0.101	0.137	−0.358	0.201	−0.059	0.130	−0.321	0.205
VA benefits	0.007⁎	0.001	0.005	0.010	0.236⁎	0.051	0.117	0.319	−0.293⁎	0.050	−0.399	−0.202
Frequent exercise	−0.045⁎	0.002	−0.048	−0.042	−1.220⁎	0.049	−1.336	−1.135	−9.408⁎	0.361	−10.146	−8.756
Anxious mood	0.002	0.002	−0.002	0.005	0.071	0.061	−0.033	0.212	−0.230⁎	0.060	−0.344	−0.116
Depressed mood	0.010⁎	0.002	0.006	0.014	0.297⁎	0.063	0.158	0.419	−0.455⁎	0.060	−0.570	−0.331
Daily tobacco	−0.006⁎	0.003	−0.011	−0.001	−0.162⁎	0.077	−0.339	−0.035	−0.698⁎	0.077	−0.847	−0.553
Daily alcohol	−0.012⁎	0.003	−0.018	−0.006	−0.421⁎	0.126	−0.709	−0.188	−0.226	0.116	−0.459	0.001
Service Era (Ref: OIF/OEF)												
Gulf War	0.016⁎	0.002	0.013	0.019	0.417⁎	0.058	0.293	0.521	0.099	0.056	−0.014	0.207
Vietnam/Korean War	0.008⁎	0.003	0.003	0.013	0.290⁎	0.089	0.142	0.493	−0.051	0.087	−0.218	0.121
Employment status (Ref: Employed)												
Retired	<0.001^g^	0.003	−0.005	0.006	−0.004	0.090	−0.175	0.178	−0.061	0.089	−0.233	0.119
Disabled	0.011 ⁎	0.004	0.004	0.018	0.223⁎	0.098	0.044	0.444	−0.544⁎	0.103	−0.748	−0.346
Unemployed	−0.003	0.002	−0.008	0.001	−0.076	0.069	−0.206	0.073	−0.324⁎	0.066	−0.457	−0.204
Survey year	0.003	0.001	−0.001	0.005	0.077	0.046	−0.032	0.154	−0.027	0.046	−0.115	0.061

a Regional categories were coded as dummy variables. Because the Pacific region had the lowest average BMI and obesity prevalence, it served as the reference category in the BMI and obesity models. Because the Northeast region had the lowest proportion of FE among veterans, it served as the reference category in the exercise model.

b Beta coefficient (b), standard error (SE), and confidence interval (CI) Bootstrapped with 1999 resamples.

c BMI was transformed with Log_10_.

d Model type: Robust linear regression.

e Model type: ML logistic regression.

f Model type: ML logistic regression.

g Precise estimate = 0.0004.

⁎ Statistically significant variable based on 95% bias corrected and accelerated (BCa) CI.

The overall OB model (Table 2) was statistically significant, *x*^*2*^_(9731)_ = 10,850, *p* < 0.001. All regions except the Northwest had significantly higher proportions of OB than the Pacific region. Cohen's *h* was calculated for the aforementioned statistically significant regional differences to determine the practical difference in proportions: Southcentral vs. Pacific, Cohen's *h* = 0.146; Southeast vs. Pacific, Cohen's *h* = 0.144; Midwest vs. Pacific, Cohen's *h* = 0.133; Northeast vs. Pacific, Cohen's *h* = 0.089; Mid-Atlantic vs. Pacific, Cohen's *h* = 0.061. Covariates associated with greater likelihood of OB included being male, having VA healthcare benefits, infrequent exercise, having depressed mood, not using alcohol/tobacco daily, having served in the Gulf/Vietnam/Korean war era, and having a disability.

The overall FE model (Table 2) was statistically significant, *x*^*2*^_(9882)_ = 11,291, *p* < 0.001. The Pacific, Southeast, and Mid-Atlantic regions had higher proportions of FE than the Northeast region. Cohen's *h* was calculated for the aforementioned statistically significant regional differences to determine the practical difference in proportions: Mid-Atlantic vs. Northeast, Cohen's *h* = 0.084; Pacific vs. Northeast, Cohen's *h* = 0.078; Southeast vs. Northeast, Cohen's *h* = 0.046. Covariates associated with greater likelihood of FE included being male, not having VA health care benefits, having a lower BMI, not reporting symptoms consistent with anxious/depressed mood, not using tobacco daily, being employed, and not having a disability.

## 4 Discussion

Results of this study showed that veteran BMI, obesity prevalence, and engagement in FE varied by U.S. region, although effect sizes for differences were small and veterans in all regions demonstrated an average BMI within the overweight BMI category. Outcomes of multivariate regression models showed that high BMI and high obesity prevalence were concentrated in the Midwestern and Southern regions, which corresponds to studies of the general population (Myers, 2015). Furthermore, results showed that FE was most prevalent in the Mid-Atlantic region, but ranged from 63.3% to 67.0% in other regions.

Several covariates in the present study exhibited significant associations with our outcomes. Specifically, depressed/anxious mood, risky health behaviors (e.g., smoking), and a service-connected disability were associated with higher BMI, greater likelihood of obesity, and reduced likelihood of FE. These results correspond to other studies of the veteran population (Almond et al., 2008; Breland et al., 2017; McDaniel et al., 2017; Teeters et al., 2017; Trivedi et al., 2015).

When considering the findings of this report, several limitations must be acknowledged. Secondary analysis of survey data, while providing a large sample of new Team RWB respondents, limited the scope of questions asked. Data were self-reported, which could be problematic due to respondent recall or reluctance to truthfully answer sensitive questions (e.g., weight, depressed mood). Further, regarding this point, it is possible that what constitutes FE could vary from person to person. Some research has shown that differences in self-report and objective measures of physical activity and medical conditions differ among military veterans (de la Motte et al., 2018; Smith et al., 2008).

The items assessing anxious/depressed mood were broad (Thomas et al., 2015). As a result, the present study's prevalence rates indicate symptom self-report at a broad range of severity levels. Respondents self-selecting membership in a service organization with an emphasis on physical fitness may be different than veterans in the larger subpopulation. Though risk for obesity in the RWB respondent pool is similar to civilians, the present study may underestimate the conditions in the larger veteran community.

## 5 Conclusion

This study is the first to nationally evaluate regional differences in veteran BMI/obesity. It provides important information for the military-connected community. Team RWB was designed to implement physical activities to improve health outcomes in transitioning service members. Their national presence and ability to create engaging physical activities for veterans addresses a critical need. In geographic locations where chapters do not exist, other public health programs aimed at reducing risk for obesity and increasing exercise frequency among the general population could be tailored to target military veterans. Additionally, information about obesity risk in specific Team RWB regions is useful to the organization. Programming to promote supported physical activity programs (especially for disabled veterans), promote mental well-being, and decrease negative health behaviors around smoking and drinking are especially warranted.
